# Factors Influencing Suicidal Ideation and Attempts among Older Korean Adults: Focusing on Age Discrimination and Neglect

**DOI:** 10.3390/ijerph18041852

**Published:** 2021-02-14

**Authors:** Young Ko, Song Yi Han, Hye-Young Jang

**Affiliations:** 1 College of Nursing, Gachon University, Incheon 21936, Korea; moodory@gmail.com; 2 Department of Nursing Science, Sunmoon University, Asan-si 31460, Korea; syhan@sunmoon.ac.kr; 3 College of Nursing, Hanyang University, Seoul 04763, Korea

**Keywords:** older adults, suicidal ideation, suicide attempt, age discrimination, neglect

## Abstract

This study was conducted to identify factors influencing the development of suicidal ideation and the transition from suicidal ideation to attempts by focusing on experiences of age discrimination and neglect among older Korean adults. This study analyzed data from 10,042 older adults from the 2017 National Survey of Older Koreans using national representative samples. Multiple logistic analyses were used to identify factors influencing the development of suicidal ideation and transition from suicidal ideation to attempts. While younger age, higher educational attainment, living alone, number of chronic diseases, depressive symptoms, social isolation, social support, experience of neglect, and age discrimination influenced the development of suicidal ideation, all of these did not influence the transition from ideation to attempts. Factors influencing this transition included male gender, less educational attainment, and experience of age discrimination. Thus, social efforts to reduce age discrimination are necessary to prevent suicide attempts among Korean older adults.

## 1. Introduction

Suicide in older adults is a major public health issue due to the rising elderly population and the persistent trend of increasing suicide rates with age. Suicide rates are highest in those aged 70 years or older for both men and women in almost all regions, even though suicide attempts are most common among adolescents [1]. In particular, the suicide rate among South Koreans aged over 65 years was 48.6 people per 100,000, which is 2.9 times higher than the average of 18.4 people per 100,000 over 65 years of age in the member countries of the Organization for Economic Cooperation and Development (OECD) [2]. Suicidal issues in later life are expected to be taken more seriously due to the aging of baby boomers in Korea. 

Older adults tend to have adverse health outcomes due to the aging process that naturally appears as they age, changes in their role in the home or society, and decreased access to health care services, etc. [3,4]. Social exclusion and discrimination against older adults have negative effects on health by limiting access to health information, health services, and the social determinants of health [4]. Older adults are vulnerable to suicide. They experience difficulties due to a decline in function, multimorbidity, age-related loss, and decreasing autonomy and sociality. This leads some older adults to think about death and suicide for a long time, and they may eventually attempt suicide [5,6,7]. Older adults who thought about or attempted suicide were more likely to have financial problems, family discord, poor social support, and limited access to medical care [7]. Notably, suicidal behaviors in older adults are more lethal than in younger age groups since older adults tend to be frailer, be more likely to live alone, and use more lethal means than younger age groups [7,8,9]. Therefore, it is important to identify older adults who are more likely to attempt suicide and implement interventions to protect them.

Many studies have found that suicidal ideation and attempts are highly related [5,6]. However, not all people who exhibit suicidal ideation attempt suicide [10,11]. Traditional risk factors for suicide, such as depression, hopelessness, psychiatric disorders, and impulsiveness predict suicidal ideation. However, they poorly predict suicide attempts among those with suicidal ideation [12,13]. Therefore, recent researchers have emphasized the importance of a better understanding of suicidal risk in the progression from suicidal ideation to action [10,14]. One theory based on an ideation-to-action framework is the interpersonal theory of suicide proposed by Joiner et al. [15]. They posit that while a thwarted belongingness and perceived burdensomeness encourages suicidal desires, a “suicide ability” acquired through physical self-harm or the experience of violence is essential to achieve suicide attempts or death due to suicide [15]. 

Several studies investigating risk factors that influence the transition from suicidal ideation to attempts have been conducted in adolescents [11] and young adults [12]. Mars et al. [11] reported that the extent of exposure to self-harm in others and the presence of psychiatric disorders differentiate adolescents who attempt suicide from those who only experience suicidal ideation. Whethrall et al. [12] reported that acquired capacity, mental imagery about death, impulsivity, and being more likely to know friends who had attempted suicide differentiated the suicide attempt group from the suicidal ideation group. It has been reported that acquired ability or volitional factors influence the transition from suicidal ideation to attempts [11,12]. However, very few studies have been conducted to identify factors influencing the transition from suicidal ideation to attempts in older adults. A study conducted on older Korean adults found that stress levels and depression influenced the transition from suicidal thoughts to attempts. However, functional limitations, multimorbidity, health behaviors, pain, and dependency in activities of daily activities did not [13]. In a study on older Chinese adults [16], depression, loneliness, and urban residence were related to suicidal ideation and attempts. However, age, financial burdens, social networks, and functional limitations were related only to suicidal ideation. Moreover, these two studies have a limitation since they do not include factors regarding the acquired capability affecting suicide attempts in the analysis. 

A possible factor influencing the transition from suicidal ideation to attempts is the experience of violence. The exposure to painful and provocative experiences over time, such as age discrimination or neglect from family members or caregivers may contribute to the development of acquired capability [17]. A meta-analytic review study reported that perceived discrimination could build up over time and eventually affect both an individual’s physical and mental health [18,19]. Age discrimination has been reported as a factor related to suicidal thoughts in Korean [20] and Chinese Americans [21]. In the past, when the traditional idea of filial piety in Confucianism was recognized as an important social norm, the older adults’ status as social adults was respected in Asian countries. However, owing to rapid changes in values and the coming of an information society, families have gradually become nuclear families and individualism has increased [22]. Older Korean adults have come to be considered socially dependent groups and thus, experience age discrimination [23]. Neglect from family members or caregivers can also influence suicide among older adults. Elderly abuse has been reported as a factor influencing suicidal ideation in Korea [23] and China [24]. In both countries, families are mainly responsible for caring for the elderly. Family discord can also lead to more severe depression and suicides among older adults than other population groups [7,25]. However, very few studies have investigated the role of age discrimination and neglect in influencing the transition from suicidal ideation to attempts among older adults. 

Studies have shown that demographics, general psychopathology, physical illness, cognitive problems, and social factors may be related to both the development of suicidal ideation and transitions from suicidal ideation to attempts [26]. Thus, in this study, we examined the characteristics of older adults who have experienced age discrimination and neglect (Aim 1) and identified factors influencing the development of suicidal ideation and the transition from ideation to suicide attempts (Aim 2). 

## 2. Materials and Methods 

### 2.1. Study Design and Participants

This study analyzed data from the 2017 National Survey of Older Koreans [27]. This survey was conducted by the Korean Ministry of Health and Welfare in 2017. The National Survey of Older Koreans has been conducted at 3-year intervals since 1988. The survey uses nationally representative samples of non-institutionalized Korean older adults aged 65 or over who lived in the community. The appropriate sample size was calculated using the 2010 population and housing census data. A nationwide probability sample of older adults was selected using a stratified two-stage cluster sample design. The calculated sample considered people from 16 regions (seven metropolitan and nine provincial regions), including both urban and rural areas [27]. A total of 10,299 subjects participated in the survey. For this study, we included a total of 10,042 participants with no missing data in main variables in the analysis.

### 2.2. Measure

#### 2.2.1. Suicidal Ideation and Suicide Attempts

Suicidal ideation and suicide attempts were assessed using the following question: “Have you thought about ending your life through any method in the past year?” If suicidal ideation was present, a question regarding suicide attempts was asked as follows: “Have you ever tried to attempt suicide specifically in the past year?” They were asked to respond, “yes” or “no.” For this study, participants who reported no suicidal ideation were included in the “no suicidal ideation” group. Those who reported suicidal ideation, but no suicide attempts were included in the “suicidal ideation only” group. Those who thought about and attempted suicide were included in the “suicide attempt” group. 

#### 2.2.2. Risk Factors 

Demographics were assessed by asking about participant gender, age, educational attainment, employment status, annual household income, and living arrangements. The participants were categorized based on age into two groups (65–74 and 75 years and older). The annual household income was classified according to quartile distributions (lowest 25%, 25–50%, 51–75%, highest 25%). Living arrangements were categorized into two groups (living alone or living with others). 

Depressive symptoms were assessed using 15 items from the Geriatric Depression Scale (GDS) developed by Sheikh and Yesavage [28]. They were translated into Korean, and their validity was evaluated by Kee [29]. Each GDS item was assessed by “yes” or “no,” and the total depressive score ranged from 0 to 15. A score of 4 or lower is categorized as “normal,” and scores of 5–9 are categorized as “mild depression.” Scores of 10–15 are classified as “severe depression.” In this study, a participant was considered to have depressive symptoms if their score was 10 or higher. The scale in the study showed good reliability (Cronbach’s alpha = 84).

Physical illness was assessed by the number of chronic diseases and dependence in activities of daily living.

We assessed self-reported diagnosed chronic diseases (including hypertension, stroke, hyperlipidemia, angina, myocardial infarction, diabetes, thyroid disease, arthritis, neuropeptide, osteoporosis, back pain, chronic bronchitis, emphysema, asthma, tuberculosis, cataracts, glaucoma, chronic otitis media, cancer, gastroduodenal ulceration, hepatitis, liver cirrhosis, chronic kidney disease, benign prostatic hyperplasia, urinary incontinence, sexually transmitted diseases, anemia, skin disease, depression, dementia, fracture, insomnia, Parkinson’s disease, etc.). 

Dependence in activities of daily living was defined as the need for help with personal care in at least one item on the Korean Instrument Activities of Daily Living (K-IDL) or Korean Activity of Daily Living (K-ADL) [30]. 

The cognitive function was measured using the Mini-Mental State Examination for Dementia Screening (MMSE-DS) developed by Kim et al. [31]. The validity and reliability of this instrument were evaluated [31]. This instrument consisted of 19 items, and the scores were classified into normal and cognitive decline according to the criteria for cognitive decline by gender, age, and education level. Cronbach’s alpha in the present study was 81.

Social factors were assessed for the experience of violence (age discrimination and neglect from family members or caregivers), social isolation, and social support.

The experience of age discrimination was assessed via the following question: “Have you ever experienced discrimination in your daily life because you are an older adult, such as while using public transportation, restaurants or coffee shops, markets or department stores, public institutions, medical institutions, or the workplace?” The participants were asked to answer “yes” or “no”. 

We defined neglect committed by family members or caregivers during the last 12 months. Neglect was assessed via the following two questions: “Does someone in your family member or caregiver not care (e.g., no nursing, no hygiene, no meals) for you when you are sick?” and “Does someone from your family members or caregivers not visit you or offer you a living allowance?” Those who answered “yes” in one of the two items were considered to have experienced neglect.

Social isolation was created by combining the frequency of contact with family and contact with friends, neighbors, and acquaintances [32]. The frequency of contact with family or friends, neighbors, and acquaintances was measured via the following two questions: “How often do you make contact over the telephone, text messaging, e-mail, and letters with your family, including spouse, children, and grandchildren who do not live with you?” This same question was also asked for relatives, friends, neighbors, and acquaintances. Two items are assessed on a 7-point scale (1 = “never”, 2 = “1–2 times a year”, 3 = “1–2 times every 3 months”, 4 = “1–2 times a month”, 5 = “at least once a week”, 6 = “2–3 times a week”, 7 = “nearly every day (more than 4 times a week)”). Both questions were recorded by combining the response categories: Not isolated (nearly every day (more than 4 times a week), 2–3 times a week, at least once a week, 1–2 times a month) and isolated (1–2 times every 3 months, 1–2 times a year, or never). We combined the two dichotomous variables with two categorical patterns of social isolation variables: (1) Subjectively isolated from family, relatives, friends, neighbors, and acquaintances, and (2) not subjectively isolated from family, relatives, friends, neighbors, and acquaintances. Social support was assessed by asking the question, “How many close friends, relatives, and acquaintances do you have (people with whom you can talk about what is on your mind)?”.

### 2.3. Data Collection

Trained interviewers collected the data. We used publicly available data that did not include identification information. We requested data from the 2017 National Survey of Older Koreans through the public data portal site (https://www.data.go.kr/ (accessed on 10 February 2021)) [19]. The data were provided after reviewing our application. The protocol for secondary analysis via the use of these data was approved by the Investigational Review Board of the university with which the researchers were affiliated (IRB no. 1044396-202009-HR-174-01).

### 2.4. Statistical Analysis

Descriptive statistics were used to identify the characteristics of demographics, general psychopathology, physical illness, cognitive problems, and social factors, and suicidal ideation and attempts among the participants. The characteristics of older adults who had experienced age discrimination and neglect were identified using chi-square tests, the t-test, or ANOVA (Aim 1). 

To investigate the respective influence of the variables, initial univariate regression analyses were conducted. In order to identify which variables independently distinguished the groups, two multiple logistic regressions were performed: One with the “no suicidal ideation” group as the reference, and the other with the “suicidal ideation only” group as the reference (Aim 2). 

For the multiple logistic analyses, we entered 14 risk factors (gender, age, educational attainment, household income, employment, living alone, depressive symptoms, dependency in activities of daily living, number of chronic diseases, cognitive decline, experience of age discrimination, experience of neglect, social isolation, and social support) into the models since they have been related to suicidal ideation and attempts in the literature [26]. Odds ratios (OR) indicated the likelihood of membership in the “suicidal ideation only” group (relative to the “no suicidal ideation” group and the “suicide attempt” group (relative to the “suicidal ideation only” group). 

## 3. Results

### 3.1. Characteristics of the Participants and Experience of Age Discrimination

The characteristics of the participants are shown in Table 1. About 43% were male and 57% were female, and the average age was 73.9 (±6.5) years. About 24% of the older adults lived alone and 31% were employed. In the overall sample of 10,042 participants, 9372 (93.3%) reported no history of thought or attempts (“no suicidal ideation” group), 581 (5.8%) reported suicidal thoughts (“suicidal ideation only” group), and 89 (0.9%) reported suicide attempts (“suicide attempt” group). 

The prevalence of experience of age discrimination was 5.4%. Older adults who were male, were older, had lower household income, lived alone, and experienced age discrimination more than their counterparts (*p* < 0.05). Older adults who had depressive symptoms, had chronic diseases, reported more neglect, those who were more socially isolated or had less social support reported more age discrimination (*p* < 0.05). Suicidal ideation and attempts were significantly related to the experience of age discrimination (*p* < 0.001) (Table 1).

### 3.2. Experience of Neglect from Family Members or Caregivers

The prevalence of neglect from family members or caregivers was 2.6%. Older adults who had lower educational attainment had lower household income, were unemployed, and lived alone reported more experience of neglect (*p* < 0.001). Older adults with depressive symptoms, more chronic diseases, or with dependency in activities of daily living reported more experiences of neglect (*p* < 0.001). Those who were socially isolated or had less social support also reported more experiences of neglect (*p* < 0.001). Suicidal ideation and suicide attempts were significantly related to neglect (*p* < 0.001) (Table 2).

### 3.3. Factors Influencing Suicidal Ideation and Suicide Attempts

#### 3.3.1. Factors Influencing the Development of Suicidal Ideation in the Past Year

The results of the univariate and multivariate logistic analyses identifying factors that influenced the development of suicidal ideation in the past year are shown in Table 3. 

Results of the univariate logistic analysis indicated that the “suicidal ideation only” group differed significantly from the “no suicidal ideation” group in terms of demographics (gender, age, educational attainment, household income, unemployment, and living alone), depressive symptoms, physical illness (number of chronic diseases and dependency in the activities of daily living), cognitive decline, and social factors (experience of age discrimination and neglect, social isolation, and social support).

Results of multiple logistic analysis indicated that the “suicidal ideation only” group differed significantly from the “no suicidal ideation” group in terms of demographics (age, educational attainment, and living alone), depressive symptoms, physical illness (number of chronic disease), and social factors (experience of age discrimination and neglect, social isolation, and social support). Compared to older adults who did not think of suicide, older adults who were more likely to think of suicide were less than 74 years old (adjusted OR (aOR) = 0.59, 95% CI = 0.48–0.72)), had more than those with a higher educational attainment (aOR = 1.03, 95% CI = 1.00–1.05), lived alone (aOR = 1.27, 95% CI = 1.00–1.62), had more chronic diseases (aOR = 1.22, 95% CI = 1.16–1.28), had depressive symptoms (aOR = 5.03, 95% CI = 4.09–6.18), experienced age discrimination (aOR = 2.36, 95% CI = 1.79–3.12), experienced neglect (aOR = 2.38, 95% CI = 1.68–3.37), were socially isolated (aOR = 1.70, 95% CI = 1.40–2.07), and had more social support (aOR = 1.07, 95% CI = 1.03–1.11).

#### 3.3.2. Factors Influencing the Transition from Ideation to Suicide Attempts in the Past Year

The results of the univariate and multivariate logistic analyses identifying the factors influencing the transition from ideation to suicide attempts in the past year are shown in Table 4. 

Results of the univariate logistic analysis indicated that the “suicide attempt” group differed significantly from the “suicidal ideation only” group in terms of demographics (gender, educational attainment, and household income), and social factors (experience of age discrimination and social support).

Results of the multiple logistic analysis indicated that the “suicide attempt” group differed significantly from the “suicidal ideation only” group in terms of demographics (gender and educational attainment) and social factors (experience of age discrimination). Compared to older adults who thought about, but did not attempt suicide, older adults who were more likely to think about and attempt suicide were male (aOR = 1.82, 95% CI = 1.10–3.02), had fewer educational attainment, (aOR = 0.94, 95% CI = 0.89–0.99), and experienced age discrimination (aOR = 2.03, 95% CI = 1.16–3.55).

## 4. Discussion

This study was conducted to identify the characteristics of older adults who have experienced age discrimination and neglect and the factors influencing the development of suicidal ideation and the transition from ideation to suicide attempts in the past year using a nationally representative sample. We have shown that the factors influencing the transition from ideation to attempt were male sex, educational attainment, and the experience of age discrimination. Not all risk factors that influence the development of suicidal ideation influence the transition from ideation to attempt. The differences in factors influencing the development of suicidal ideation and progression from ideation to attempts in older adults are rarely reported in the literature, making this a unique study.

Suffering from multiple chronic diseases could cause older adults to be depressed and force them to think seriously about the value of their existence [33]. Additionally, the experience of neglect from family members or caregivers and social isolation may make them suffer due to a lack of social belonging. These results are consistent with previous studies in which depression and thwarted belongingness are the main predictors of suicidal ideation [16,23,33,34].

The experience of age discrimination in older adults’ daily lives seems to have become a consistent and painful experience. Several studies [35,36] show that age discrimination can lead to self-harm through multiple mechanisms, including decreased self-esteem, reduced sense of belonging, decreased participation in healthy behaviors, and reduced access to opportunities for upward mobility. One of the main factors causing progression from ideation to attempts is the acquired capacity for suicide proposed by three representative theories within the ideation to action framework [14]. This acquired capacity is developed and increased through experiences with painful and provocative events that increase one’s tolerance for pain, injury, and death. Over time, this leads to a higher capacity for a suicide attempt. Therefore, it is possible that the experience of age discrimination in older Korean adults is one of the experiences to influence the development of the acquired ability of suicide attempts. However, there are few studies on the effects of age discrimination on suicide attempts in Western countries other than Asian countries. In addition, other experiences related to pain, injury, fear, and death that may lead to a higher capacity for suicide attempts [8,14] were not considered in this study. It is difficult to identify how the experience of age discrimination affects the development of acquired ability, so it is necessary to reconfirm the results of this study. The necessity of mental health care, such as depression, has been suggested as an intervention to prevent suicide attempts. The necessity for intervention in cases where older adults experience age discrimination to block progression from suicidal ideation to attempt is demonstrated in this study.

A total of 5.8% of older adults experienced age discrimination in their daily lives. This is lower than the rate of age discrimination reported in European countries [37,38,39] and the US [40]. The reason why age discrimination is lower in Korea may be due to the traditional Confucian value of respecting older adults. In England, 36.8% of older adults experience age discrimination and reported that they were treated with less courtesy (17.7%) [38]. It was reported that older adults in the UK had more experience of age discrimination than older adults in Korea, but older adults in Korea have more suicidal ideation than those in the UK [39]. However, it is difficult to directly compare the rate of age discrimination in this study with the results of other studies. One reason is that various tools have been used in previous studies [37,38]. Another reason is that the national context also affects the level of ageism experienced among older adults [41]. A study has shown differences in the degree of age discrimination among countries due to modernization, employment, and culture for older adults [37,38,39,40,41]. Nevertheless, it is consistent that age discrimination negatively affects older adults’ mental health in previous studies [37,40,42,43], especially in poor older adults and less-developed countries [37]. We found that this is a strong factor influencing suicide attempts in older Korean adults. Thus, social efforts to reduce age discrimination are needed.

A total of 2.4% of the older adults reported that they experienced neglect from family members or caregivers. These experiences have influenced older adults to think about suicide. It seems that the reporting rate of neglect was quite low in Korea when compared to that of foreign countries [44,45]. Elder neglect is likely to go underreported for several reasons [45]. In this study, since the interviewers visited older adults’ homes to ask the questionnaires for this study, older adults with family members or caregivers may have difficulty talking about their neglect experience due to shame and fear of retribution. As the number of older adults in need of care increases, we expect the issue of neglect to become a more pressing concern. Efforts should be made to improve awareness and increase reporting rates.

We found that females thought more about suicide than males, but males made more suicide attempts than females in this study. These results are consistent with those of previous studies that female older adults have more suicidal thoughts than male older adults [46,47]. These results are also consistent with those of a previous study using the data from eight European countries that showed a higher rate of fatal suicide attempts and mortality in males than in females [48].

While household income was not related to suicide attempts, it was related to suicidal ideation. Older adults with low socioeconomic status were more likely to experience age discrimination and neglect. Despite inconsistent findings regarding the association between the education level and experience of age discrimination [21,41,42] in the literature, the socioeconomic status is associated with age discrimination and neglect. Thus, healthcare providers should monitor the accessibility of mental health care services to support vulnerable populations.

Older adults who live alone, are socially isolated, or have poor social support had greater experiences of age discrimination and neglect. Thus, there were more instances of these adults thinking about and attempting suicide. This is consistent with the results of previous studies that show living alone and social isolation to be related to suicidal ideation and behaviors [49], especially among male in Korea [50]. Furthermore, it is consistent with the results of some studies reporting that social support mediates or moderates the relationship between the experience of age discrimination and mental health [39]. These results suggest that screening older adults for social isolation, living arrangements, and social support, especially males, may create a point of intervention to prevent suicide attempts.

Several limitations need to be considered in the interpretation of these study results. First, this was a cross-sectional study. Thus, the causal relationship between the experience of age discrimination and transition from ideation to suicide attempts cannot be confirmed. Second, most of the independent variables were surveyed on a categorical or dichotomous scale rather than a continuous scale to collect data from a large sample. Therefore, although the independent variables include both stable time-invariant variables and time-variant variables, an analysis model considering the relationship of independent variables could not be constructed. Third, in this study, only data on suicidal ideation and attempts that occurred over the past year were collected and used. Therefore, we could not exclude participants who might had previously thought about or attempted suicide, but who had not thought about or attempted it in the past year, or those who had thought about suicide in the past year, but who might have attempted before that. Fourth, despite the experience of age discrimination in daily life, the multi-dimensionality of age discrimination [37,38] might have not been adequately measured in this study. Fifth, we used two questions to access neglect, which indicated an unsatisfied need for older adults from family members or caregivers. However, older adults may have experienced neglect, but received help from someone other than family members or caregivers. Finally, as a secondary analysis, this study did not include family history, impulsivity, alcohol and substance use, personality traits, prior self-injurious behaviors, and exposure to self-injurious behaviors that influence suicidal ideation and attempts [26]. 

## 5. Conclusions

This study found different factors influencing the development of suicidal ideation and transition from suicidal ideation to attempt. The factors influencing the transition from ideation to attempt were male sex, educational attainment, and the experience of age discrimination.

Based on this result, we suggest the following implications. First, we found differences in factors influencing suicidal ideation and attempts. Thus, these differences must be considered while developing programs to prevent suicide. Second, the experience of age discrimination is a risk factor in older adults showing suicidal ideation and suicide attempts. Thus, social efforts to reduce age discrimination in older adults are necessary. Third, it was confirmed that older adults who live alone and are socially isolated had greater experiences of age discrimination and neglect from family members or caregivers. It can be concluded that these adults thought about suicide more often. It is urgent to build a support system for older adults who do not have a family or social support system. Finally, an in-depth study to measure the multi-dimensionality of age discrimination is needed.

## Figures and Tables

**Table 1:** Experience of age discrimination. *N* = 10,042.

Variables	Category (Range)	Experience of Age Discrimination			χ^2^ or t (*p*)
		Total	No	Yes	
		*n* (%) or M ± SD	*n* (%) or M ± SD	*n* (%) or M ± SD	
Total		10,042 (100.0)	9504 (94.6)	538 (5.4)	
Gender	Male	4278 (42.6)	4026 (94.1)	252 (5.9)	4.12 (0.041)
	Female	5764 (57.4)	5478 (95.0)	286 (5.0)	
Age (years)	(65–106)	73.9 ± 6.5	73.8 ± 6.5	75.0 ± 6.6	−4.09 (<0.001)
Educational attainment (years)	(0–22)	7.2 ± 4.6	7.5 ± 4.5	7.2 ± 4.6	−1.82 (0.069)
Household income (unit: KRW 1000 won)	Q1 (30–1123)	2529 (25.2)	2365 (93.5)	164 (6.5)	15.39 (0.002)
	Q2 (1124–1888)	2515 (25.0)	2369 (94.2)	146 (5.8)	
	Q3 (1889–3323)	2505 (24.9)	2380 (95.0)	125 (5.0)	
	Q4 (3324–31500)	2493 (24.8)	2390 (95.9)	103 (4.1)	
Employment	Yes	3115 (31.0)	2935 (94.2)	180 (5.8)	1.67 (0.197)
	No	6927 (69.0)	6570 (94.8)	357 (5.2)	
Living alone	Yes	2405 (23.9)	2228 (92.6)	177 (7.4)	25.33 (<0.001)
	No	7637 (76.1)	7277 (95.3)	360 (4.7)	
Depressive symptoms	(0–15)	4.2 ± 3.6	4.1 ± 3.6	5.7 ± 4.0	−9.76 (<0.001)
Dependency in activities of daily living	Yes	2358 (23.5)	2226 (94.4)	132 (5.6)	0.380 (0.538)
	No	7684 (76.5)	7278 (94.7)	406 (5.3)	
Number of chronic diseases	(0–14)	2.7 ± 1.8	2.7 ± 1.8	3.2 ± 2.1	−6.29 (<0.001)
Cognitive decline	Yes	1681 (16.7)	1588 (94.5)	93 (5.5)	0.12 (0.728)
	No	8361 (83.3)	7916 (94.7)	445 (5.3)	
Experience of neglect	Yes	260 (2.6)	226 (86.9)	34 (13.1)	31.78 (<0.001)
	No	9782 (97.4)	9279 (94.9)	503 (5.1)	
Social isolation	Yes	2482 (24.7)	2312 (93.2)	170 (6.8)	14.68 (<0.001)
	No	7560 (75.3)	7193 (95.1)	367 (4.9)	
Social support (number of people)	(0–30)	2.3 ± 2.7	2.3 ± 2.7	2.0 ± 2.3	2.72 (0.007)
Suicidal related behaviors	No ideation	9372 (93.3)	8937 (95.4)	435 (4.6)	164.21 (<0.001)
	Suicidal ideation only	581 (5.8)	501 (86.2)	80 (13.8)	
	Suicide attempt	89 (0.9)	66 (74.2)	23 (25.8)	

Note: M ± SD (Mean ± standard deviation).

**Table 2:** Experience of neglect. *N* = 10,042.

Variables	Category (Range)	Experience of Neglect			χ^2^ or t (*p*)
		Total	No	Yes	
		n or M ± SD	n (%) or M ± SD	n (%) or M ± SD	
Total		10,042	9783 (97.4)	259 (2.6)	
Gender	Male	4278	4169 (97.5)	109 (2.5)	0.05 (0.823)
	Female	5764	5613 (97.4)	151 (2.6)	
Age (years)	(65–106)	73.9 ± 6.5	73.8 ± 6.5	74.2 ± 6.0	−0.98 (0.328)
Educational attainment (years)	(0–22)	7.2 ± 4.6	7.2 ± 4.6	5.4 ± 4.6	6.44 (<0.001)
Household income (unit: KRW 1000 won)	Q1 (30–1123)	2529	2387 (94.4)	142 (5.6)	151.98 (<0.001)
	Q2 (1124–1888)	2515	2443 (97.1)	72 (2.9)	
	Q3 (1889–3323)	2505	2474 (98.8)	31 (1.2)	
	Q4 (3324–31,500)	2493	2479 (99.4)	14 (0.6)	
Employment	Yes	3115	3054 (98.0)	61 (2.1)	7.13 (<0.001)
	No	6927	6728 (97.1)	199 (2.9)	
Living alone	Yes	2405	2258 (93.9)	147 (6.1)	157.21 (<0.001)
	No	7637	7525 (98.5)	112 (1.5)	
Depressive symptoms	(0–15)	4.2 ± 3.6	4.1 ± 3.5	7.8 ± 4.2	−5.46 (<0.001)
Dependency in activities of daily living	Yes	2358	2269 (96.2)	89 (3.8)	17.51 (<0.001)
	No	7684	7514 (97.8)	170 (2.2)	
Number of chronic diseases	(0–14)	2.7 ± 1.8	2.7 ± 1.8	3.4 ± 2.1	−5.46 (<0.001)
Cognitive decline	Yes	1681	1640 (97.6)	41 (2.4)	0.16 (0.691)
	No	8361	8143 (97.4)	218 (2.6)	
Social isolation	Yes	2482	2319 (93.4)	163 (6.6)	206.83 (<0.001)
	No	7560	7463 (98.7)	97 (1.3)	
Social support (number of people)	(0–30)	2.3 ± 2.7	2.3 ± 2.7	0.9 ± 1.3	16.50 (<0.001)
Suicidal related behaviors	No ideation	9372	9182 (98.0)	190 (2.0)	174.71 (<0.001)
	Suicidal ideation only	581	524 (86.5)	57 (9.8)	
	Suicide attempt	89	77 (86.5)	12 (13.5)	

Note: M ± SD (mean ± standard deviation).

**Table 3:** Factors associated with suicidal ideation.

Variables (Reference)	Suicidal Ideation Only (vs. No Suicidal Ideation ^a^)			
	Crude OR (95% CI)	*p*	Adjusted OR (95% CI)	*p*
Male (female)	0.80 (0.68–0.96)	0.013	0.99 (0.81–1.21)	0.912
Age, ≥75 years (65–74 years)	0.83 (0.70–0.99)	0.040	0.59 (0.48–0.72)	<0.001
Educational attainment (years)	0.98 (0.96–0.99)	0.017	1.03 (1.00–1.05)	0.028
Household income Q1 (Q4)	2.31 (1.82–2.93)	<0.001	1.31 (0.96–1.77)	0.085
Household income Q2 (Q4)	1.22 (0.94–1.59)	0.140	0.90 (0.68–1.20)	0.471
Household income Q3 (Q4)	1.19 (0.91–1.55)	0.215	1.05 (0.79–1.38)	0.758
Unemployed (employed)	1.34 (1.11–1.63)	0.003	0.84 (0.67–1.03)	0.098
Living alone (living with others)	1.99 (1.67–2.38)	<0.001	1.27 (1.00–1.62)	0.046
Depressive symptoms (no symptom)	6.30 (5.25–7.57)	<0.001	5.03 (4.09–6.18)	<0.001
Number of chronic diseases	1.33 (1.28–1.38)	<0.001	1.22 (1.16–1.28)	<0.001
Dependency in activities of daily living (no dependency)	1.63 (1.36–1.95)	<0.001	0.95 (0.76–1.19)	0.661
Cognitive decline (normal)	1.33 (1.08–1.63)	0.008	1.16 (0.93–1.46)	0.198
Experience of neglect (no experience)	5.28 (3.88–7.18)	<0.001	2.38 (1.68–3.37)	<0.001
Experience of age discrimination (no experience)	3.29 (2.55–4.25)	<0.001	2.36 (1.79–3.12)	<0.001
Social isolation (not isolated)	2.60 (2.19–3.09)	<0.001	1.70 (1.40–2.07)	<0.001
Social support (number of people)	0.96 (0.92–0.99)	<0.001	1.07 (1.03–1.11)	<0.001

Note: OR: Odds ratio; CI: Confidence interval; household income (unit: KRW 1000 won): Q1 = 30–1123, Q2 = 1124–1888, Q3 = 1889–3323, Q4 = 3324–31500; ^a^ no suicidal ideation group as a reference.

**Table 4:** Factors associated with suicide attempt.

Variables (Reference)	Suicide Attempt (vs. Suicidal Ideation Only ^a^)			
	Crude OR (95% CI)	*p*	Adjusted OR (95% CI)	*p*
Male (female)	1.44 (0.92–2.26)	0.113	1.82 (1.10–3.02)	0.020
Age, ≥75 years (65–74 years)	1.09 (0.75–1.59)	0.648	1.04 (0.63–1.71)	0.889
Educational attainment (years)	0.94 (0.90–0.99)	0.023	0.94 (0.89–0.99)	0.031
Household income Q1 (Q4)	1.77 (0.85–3.70)	0.127	1.26 (0.52–3.08)	0.610
Household income Q2 (Q4)	2.22 (1.02–4.81)	0.044	1.92 (0.85–4.35)	0.117
Household income Q3 (Q4)	1.18 (0.50–2.78)	0.712	1.11 (0.45–2.72)	0.819
Unemployed (employed)	1.19 (0.70–2.03)	0.521	1.35 (0.74–2.45)	0.324
Living alone (living with others)	1.35 (0.86–2.12)	0.191	1.26 (0.68–2.32)	0.468
Depressive symptoms (no symptoms)	1.27 (0.76–2.12)	0.354	1.00 (0.57–1.75)	0.999
Number of chronic diseases	1.06 (0.96–1.18)	0.255	1.05 (0.94–1.18)	0.368
Dependency in activities of daily living (no dependency)	1.26 (0.79–1.99)	0.337	0.93 (0.54–1.59)	0.776
Cognitive decline (normal)	0.83 (0.46–1.47)	0.516	0.87 (0.47–1.64)	0.676
Experience of neglect (no experience)	1.38 (0.71–2.71)	0.344	0.93 (0.44–1.98)	0.857
Experience of age discrimination (no experience)	2.13 (1.25–3.63)	0.005	2.03 (1.16–3.55)	0.013
Social isolation (not isolated)	1.15 (0.73–1.80)	0.546	0.89 (0.53–1.50)	0.669
Social support (number of people)	0.86 (0.77–0.97)	0.012	0.88 (0.77-1.00)	0.052

Note: OR: Odds ratio; CI: Confidence interval; household income (unit: KRW 1000 won): Q1 = 30–1123, Q2 = 1124–1888, Q3 = 1889–3323, Q4 = 3324–31,500; ^a^ suicidal ideation only group as a reference.

## Data Availability

The data that support the findings of this study are available from the corresponding author, upon reasonable request.
